# 晚期肺腺癌患者一线治疗前后*EGFR*基因突变差异性分析

**DOI:** 10.3779/j.issn.1009-3419.2018.11.03

**Published:** 2018-11-20

**Authors:** 慧 李, 时 严, 显红 刘, 影 柳, 丽霞 马, 莹 王, 岩 刘, 颖 程

**Affiliations:** 1 130012 长春，吉林省肿瘤医院肿瘤转化医学实验室 Translational Medical Research Lab, Jilin Provincial Cancer Hospital, Changchun 130012, China; 2 130012 长春，吉林省肿瘤医院肿瘤内科 Department of Medical Oncology, Jilin Provincial Cancer Hospital, Changchun 130012, China

**Keywords:** 肺肿瘤, 再次活检, *EGFR*检测, Lung neoplasms, Rebiopsy, EGFR testing

## Abstract

**背景与目的:**

表皮生长因子受体(epidermal growth factor receptor, EGFR)靶向治疗能够显著提高*EGFR*突变的晚期肺腺癌患者预后生存，但治疗及异质性等因素可导致初次和疾病进展时*EGFR*基因状态发生改变。为了探讨真实世界中*EGFR*基因突变在疾病进展和基线时的差异，我们开展了此项研究。

**方法:**

收集2015年1月-2017年12月在吉林省肿瘤医院进行*EGFR*基因检测的61例配对标本数据并进行分析。标本取材时间为治疗前和疾病进展时，所有标本均经病理学或细胞学证实，标本来源为肿瘤组织﹑恶性胸腔积液和血浆，患者为初治，一线接受化疗或靶向治疗，采用扩增阻滞突变系统法(Amplification Refractory Mutation System, ARMS)对29种*EGFR*基因突变进行检测。

**结果:**

初次和再次活检相比(*n*=61)，肿瘤组织、恶性胸腔积液和血浆标本所占的比例分别为90.2% *vs* 88.5%、6.6% *vs* 6.6%和3.2% *vs* 4.9%，其中标本类型前后一致的患者(*n*=50)*EGFR*突变差异率为72.0%，标本类型不一致患者(*n*=11)为36.3%；治疗前*EGFR*突变率为95.1%，治疗后为91.8%，二者的差异率为63.9%；化疗患者(*n*=13)治疗前*EGFR*突变率为69.2%，治疗后为92.3%，二者差异率为46.1%；靶向治疗患者(*n*=48)治疗前*EGFR*突变率为100%，治疗后为91.7%，二者差异率为70.8%。*EGFR*基因变化类型有4类：野生型变为突变型(4.9%)、突变消失(8.2%)、敏感突变类型互变(1.6%)和突变种类增加(49.1%)。

**结论:**

临床实践中，晚期非小细胞肺癌(non-small cell lung cancer, NSCLC)患者由于标本取材部位和类型及治疗的影响，治疗前后EGFR基因突变具有较大差异性，动态检测并明确*EGFR*基因状态，可以为临床医生选择精准的后续靶向治疗方案提供参考。

## Abstract (en)

**Background and objective:**

Epidermal growth factor receptor (EGFR)-based targeted therapy improves the survival of patients with advanced lung adenocarcinoma harboring *EGFR* mutations. However, factors including treatment or heterogeneity partly contribute to *EGFR* genetic status alteration between baseline and disease progresses (PD). The aim of this study is to compare difference of *EGFR* mutations between biopsy and rebiopsy in real world.

**Methods:**

Data from 61 paired specimens performed EGFR testing in Jilin Provincial Cancer Hospital between January 2015 and December 2017 were collected and analyzed. The specimens were collected at baseline and PD, confirmed by histology or cytology and categorized as tumor tissue, malignant pleural effusion or plasma. All patients were naive and received chemotherapy or targeted therapy as first-line treatment. Amplification Refractory Mutation System (ARMS) was used to detect *EGFR* mutations.

**Results:**

*EGFR* mutation rate in tumor tissue, pleural effusion or blood was 90.2% *vs* 88.5%, 6.6% *vs* 6.6% and 3.2% *vs* 4.9% at baseline or PD respectively and discrepancy was 72% and 36.3% for the same (*n*=50) or different (*n*=11) type of specimens. The *EGFR* mutation rate was 95.1% and 91.8% in patients before and after treatment, and the discrepancy was 63.9%, among which, 69.2% and 92.3% in chemotherapy-treated patients (*n*=13) with discrepancy to 46.1% (6/13), and 100.0% and 91.7% in EGFR-TKI-treated patients (*n*=48) with discrepancy to 70.8%. There were four types of alterations in terms of *EGFR* mutations: wild type turned into mutation (4.9%), mutation disappeared (8.2%), sensitive mutations transformed (1.6%), and new mutations appeared (49.1%).

**Conclusion:**

In real world, the *EGFR* mutation status in advanced non-small cell lung cancer (NSCLC) patients altered significantly, due to tissue resources and therapeutic approaches, implying the importance of rebiopsy and real-time detection of *EGFR* mutation, in order to provide data to guide precise strategy in the following treatment.

表皮生长因子受体(epidermal growth factor receptor, *EGFR*)突变是中国非小细胞肺癌(non-small cell lung cancer, NSCLC)患者最常见的驱动基因，针对其敏感突变采用EGFR酪氨酸激酶抑制剂(tyrosine kinase inhibitor, TKI)进行治疗已成为晚期NSCLC一线标准治疗方案^[1-3]^。经EGFR-TKI治疗8个月-14个月后多数患者发生耐药，其中约50%是EGFR外显子20出现T790M突变^[4]^，其他突变还包括*c-met*扩增、*HER-2*突变和小细胞肺癌转化等^[5]^。除了治疗药物的影响因素之外，新发病灶带来的肿瘤异质性也会造成*EGFR*基因突变的改变^[6-8]^。因此，动态检测疾病进展时*EGFR*基因突变状态对于指导患者后续治疗和实现全程精准治疗具有重要的意义。为了阐明临床实践中治疗前和疾病进展时*EGFR*基因突变的差异情况，本研究以一线治疗的晚期NSCLC患者为研究对象，对比了61例治疗前(基线)和疾病进展时配对标本的*EGFR*基因突变数据，为验证再次活检且进行分子病理检测的必要性提供参考数据。

## 1 资料与方法

### 1.1 资料来源

回顾性收集2015年1月-2017年12月在吉林省肿瘤医院进行*EGFR*基因检测的61例配对标本，分别来自于治疗前(基线)和疾病进展时，均经病理学或细胞学证实为NSCLC，所有患者均为一线治疗，治疗方案为EGFR-TKI或标准化疗。其中男性18例，女性43例；中位年龄55岁(范围34岁-79岁)，平均年龄52岁；不吸烟者患者高于吸烟患者，45例*vs* 16例；腺癌为主要病理类型，腺癌、腺鳞癌和鳞癌患者分别为63例、1例和1例；Ⅳ期、Ⅲ期和Ⅱ期患者分别为63例、1例和1例；标本类型为组织、恶性胸腔积液及血浆，其中初次和再次检测的例数分别为55例*vs*54例，4例*vs*4例，2例*vs*3例。

### 1.2 试剂与仪器

红细胞裂解液购自BD公司，*EGFR*基因检测试剂盒购自厦门艾德生物医药科技有限公司(AmoyDx)，DNeasy Blood and Tissue Kit购自QIAGEN，Nanodrop2000C购自Thermo公司，LightCycler 480购自罗氏公司。

### 1.3 DNA提取和EGFR基因扩增

按照说明书提取不同标本来源的DNA，DNA质量控制标准为浓度 > 10 ng/μL，OD_260_/OD_280_比值为1.8-2.1。按照说明书进行*EGFR*基因扩增和突变分析：每个反应管中加入模板DNA 2 ng，PCR扩增条件为95 ℃ 5 min，15×(95 ℃ 25 s, 64 ℃ 20 s, 72 ℃ 20 s)；31×(93 ℃ 25 s, 60 ℃ 35 s, 72 ℃ 20 s)，60 ℃时收集FAM和HEX信号。判定标准为：Ct值< 26为*EGFR*突变阳性，Ct值≥29为*EGFR*突变阴性，Ct值在26-29之间判断样本△Ct值，如果△Ct值小于对应的Cut-off值，样本为*EGFR*突变弱阳性。

### 1.4 疗效评价及随访

患者治疗后2个月进行首次评价，以后每1个月住院复查或门诊、电话随访。按照实体瘤近期疗效评价标准(Response Evaluation Criteria in Solid Tumors, RECIST)评估病灶变化情况，分为完全缓解(complete response, CR)、部分缓解(partial response, PR)、疾病稳定(stable disease, SD)和疾病进展(progressive disease, PD)，有效率(response rate, RR)=(CR+PR)/总例数×100%；临床获益率(clinical benefit rate, CBR)=(CR+PR+SD)/总例数×100%；所有患者均签署知情同意书。

### 1.5 统计分析

采用SPSS 17.0统计软件进行数据分析，计数资料的组间比较采用*chi-square test*检验，采用*McNemar*检验对比分析接受治疗前和接受治疗后*EGFR*基因突变状态的改变情况，采用*Cochran-Armitage*趋势检验来分析*EGFR*基因突变状态的改变同PR、SD和PD等临床治疗结果的相关性，以*P* < 0.05为差异有统计学意义。

## 2 结果

### 2.1 *EGFR*基因突变状态与临床因素相关性分析

初次活检标本中*EGFR*基因突变状态与性别有关(*P*=0.000, 3)，疾病进展时再次活检标本中*EGFR*基因突变状态与吸烟有关(*P*=0.039)，其他临床相关因素如年龄、分期等无显著相关性(*P* > 0.05)(表 1)。

**1:** 61例配对样本的患者临床相关资料 Clinical characteristics data in 61 patients with paired specimens

Item	*n*	First biopsy		*P*	Rebiopsy		*P*
		Mutation	Wild type		Mutation	Wild type	
Age (yr)				0.289			0.164
< 65	45	42	3		40	5	
≥65	16	16	0		16	0	
Gender				0.039			0.118
Male	18	15	3		15	3	
Female	43	42	1		41	2	
Smoking status				0.352			0.000
Former smoker	16	16	2		13	5	
Never smoker	45	41	2		43	0	
Stage				0.703			0.667
Ⅱa-Ⅲa	2	2	0		2	0	
Ⅲb-Ⅳ	59	55	4		54	5	
PS				0.630			0.820
0-1	52	48	3		47	4	
≥2	9	9	1		9	1	
Rebiopsy type				0.494			0.400
Tissue	54	51	4		49	5	
Liquid biopsy	7	6	0		7	0	
PS: performance status.							

### 2.2 初次和再次活检*EGFR*突变状态

初次和再次活检相比，*EGFR*突变率均有差异，且与标本类型无关，初次和再次活检相比，送检的标本种类相同，包括组织、恶性胸腔积液和血液，但各种标本所占比例有变化，分别为90.2%(55/61)*vs*88.5%(54/61)、6.6%(4/61)*vs*6.6%(4/61)和3.2%(2/61)*vs*4.9%(3/61)。两次活检标本类型相同患者的EGFR基因突变不一致率为72.0%(36/50)，两次标本不同患者EGFR基因突变不一致率为36.3%(4/11)。

治疗前*EGFR*突变率为95.1%(58/61)，突变类型主要为19Del(*n*=28)、L858R(*n*=27)和其他类型突变(*n*=3)；再次活检标本*EGFR*突变率为91.8%(56/61)，突变类型主要为19Del(*n*=12)、L858R(*n*=12)和其他类型突变(*n*=5)，其中，新出现T790M突变27例，均为伴随突变，包括T790M-19Del(*n*=15)、T790M-L858R(*n*=10)、T790M-G719X(*n*=1)。治疗前和疾病进展相比，*EGFR*基因突变不一致率为63.9%(39/61)。*EGFR*基因突变变化类型可分为4种：野生型变为突变型(*n*=3)、基因突变消失(*n*=5)、敏感突变类型转换(*n*=1，L858R和19Del相互转换1例)和突变类型增加(*n*=29，伴随T790M的复合突变27例，伴随其他类型复合突变3例)，再次活检中T790M突变最常见(44.2%)(*McNemar*检验，*P* > 0.05；表 2)。

**2:** 61例患者初次*vs*再次活检*EGFR*基因状态的改变情况 The change in *EGFR* mutation status with primary biopsy *vs* re-biopsy for 61 patients

Item	Rebiopsy		Total
	Wild type	Mutation	
Wild type	0	3	3
Mutated	5	53	58
Total	5	56	61
EGFR: epidermal growth factor receptor.			

行化疗的患者治疗前*EGFR*突变率为76.9%(10/13)，治疗后为92.3%(12/13)，二者的不一致率为38.5%(5/13)，差异类型分别为S768I转变成G719X+S768I复合突变(*n*=1)、野生型变成19Del(*n*=2)、G719X(*n*=1)、19Del变成*EGFR*野生型(*n*=1)。行靶向治疗患者治疗前EGFR突变率为100.0%(48/48)，治疗后为91.7%(44/48)，二者的差异率为70.8%(34/48)。差异类型分别为出现T790M突变(*n*=27)、*EGFR*突变型变为野生型(*n*=4)、L858R转换为19Del(*n*=1)、L858R转换为L858R+19Del复合突变(*n*=1)和19Del转换为L858R+19Del复合突变(*n*=1)。靶向治疗后*EGFR*突变状态的改变具有统计学差异(*McNemar*检验，*P*=0.000, 7；表 3)。

**3:** 48例*EGFR*突变患者一线接受靶向治疗后*EGFR*基因状态的改变情况 The change in *EGFR* mutation status after 48 patients received EGFR-TKI as first line therapy

	After EGFR-TKIs			
	T790M	Change	No change	Total
L858R	11	4	7	22
19Del	15	3	6	24
G719X	1	0	1	2
Total	27	7	14	48
TKI: tyrosine kinase inhibitor.				

### 2.3 治疗前后*EGFR*突变状态变化类型不同的患者疗效之间有差异

48例患者一线接受靶向治疗，治疗有效率(response rate, RR)为72.9%(35/48)，临床获益率(clinical benefit rate, CBR)为93.7%(45/48)；按治疗前后*EGFR*基因突变的变化形式不同分为4类：基因突变相同组、突变转化为野生型组、敏感突变类型转化组和出现T790M组，4组患者的治疗RR分别为85.7%(12/14)、25.0%(1/4)、33.3%(1/3)、77.7%(21/27)，CBR分别为100.0%(14/14)、50.0%(2/4)、100.0%(3/3)、96.3%(26/27)，4组之间的疗效差异具有显著相关性，基因突变频率同较好的靶向临床反应显著相关(*Cochran-Armitage*检验，*P*=0.026；图 1和表 4)。13例患者一线接受化疗，RR为30.7%(4/13)，CBR为76.9%(10/13)；按治疗前后EGFR基因突变的变化形式不同分为4类：基因突变相同组、突变转化为野生型组、敏感突变类型转化组和野生型转化为突变组，4组患者的治疗RR分别为37.5%(3/8)、0.0%(0/1)、100.0%(1/1)、0.0%(0/3)，CBR分别为75.0%(6/8)、100.0%(1/1)、100.0%(1/1)、66.7%(2/3)(*Cochran-Armitage*检验，*P* > 0.05；图 2和表 5)。一线接受靶向治疗的患者RR和CBR均高于一线接受化疗的患者(RR:72.9% *vs* 30.7%;CBR:93.7% *vs* 76.9%)。

**1:**
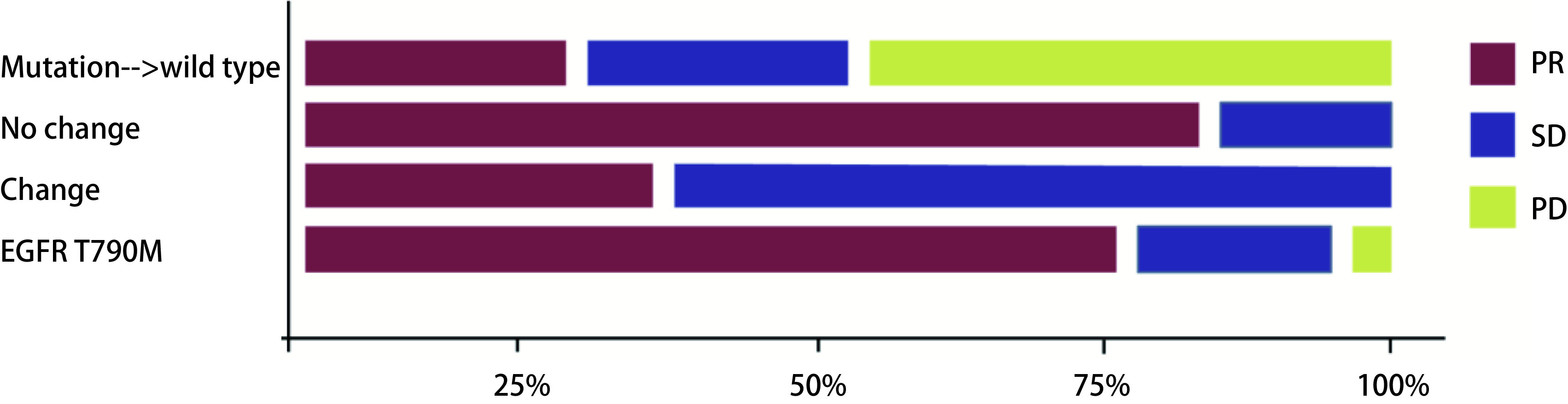
基因突变相同组﹑突变转化为野生型组、敏感突变类型转化组和出现T790M组的疗效差异 Efficacy differences in terms of the same mutations group, mutation turned into wild type group, sensitive mutations transformed group, and T790M group. PR: partial response; SD: stable disease; PD: progressive disease.

**4:** 一线靶向前后（初次*vs*再次活检）*EGFR*突变状态的改变情况和相对应的治疗疗效 The Changes in *EGFR* mutation status and corresponding therapeutic effects before and after EGFR-TKI as first line therapy (primary biopsy *vs* re-biopsy)

*EGFR* mutation change from pre- to TKI	Treatment response			
	PR	SD	PD	Total (*n*=48)
Mutation→wild type	1	1	2	4
No change	12	2	0	14
Change	1	2	0	3
EGFR T790M	21	5	1	27

**2:**
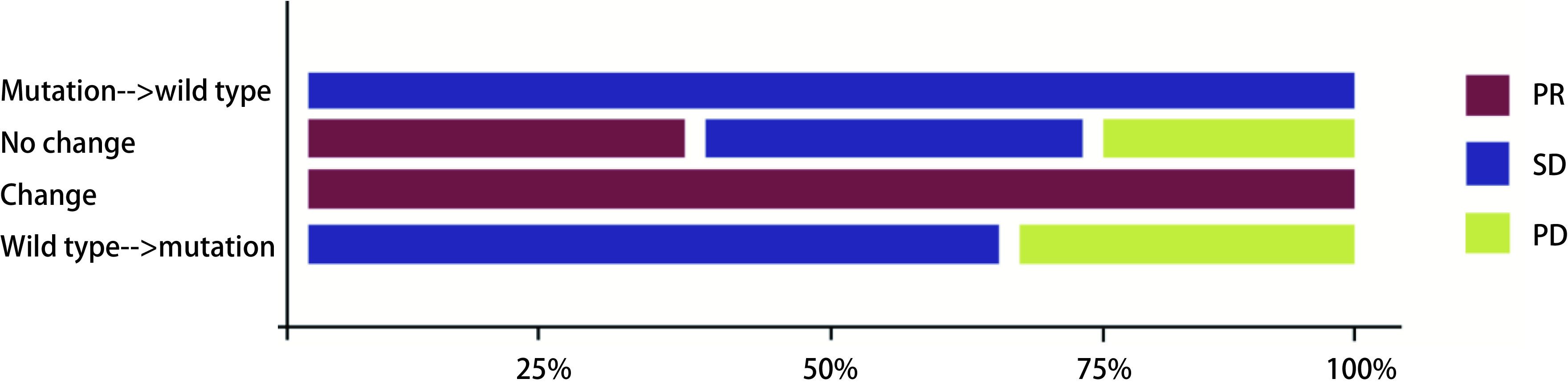
基因突变相同组﹑突变转化为野生型组、敏感突变类型转化组和野生型转化为突变组的疗效差异 Efficacy differences in terms of the same mutations group, mutation turned into wild type group, sensitive mutations transformed group, and wild type turned into mutation group

**5:** 一线化疗前后（初次*vs*再次活检）*EGFR*突变状态的改变情况和相对应的治疗疗效 The Changes in *EGFR* mutation status and corresponding therapeutic effects before and after chemotherapy as first line therapy (primary biopsy *vs* re-biopsy)

Change from pre- to post-chemotherapy	Treatment response			
	PR	SD	PD	Total (*n*=13)
Mutation→wild type	0	1	0	1
No change	3	3	2	8
Change	1	0	0	1
Wild type→mutation	0	2	1	3

### 2.4 一线靶向治疗后获得性耐药T790M阳性和阴性患者临床相关因素分析

48例患者接受一线靶向治疗，其中27例患者在二次活检中检测到T790M突变，比例为56.2%。年龄、性别、吸烟状态和组织学亚型等在内的临床因素同*EGFR* T790M突变状态之间没有相关性(*P* > 0.05)(表 6)。

**6:** 一线靶向治疗后获得性耐药T790M阳性和阴性患者临床相关因素差异 Differences in clinical relevant factors between patients with acquired drug resistance T790M positive and T790M negative after EGFR-TKI as first line therapy

Item	T790M (*n*=27)	T790M wild type (*n*=21)	*P*
Age (yr)			0.575
≤65	20	14	
> 65	7	7	
Gender			0.129
Male	4	7	
Female	23	14	
PS			0.381
0-1	22	19	
≥2	5	2	
Smoking status			0.573
Former smoker	7	4	
Never smoker	20	17	
Stage			0.251
Ⅱa-Ⅲa	0	1	
Ⅲb-Ⅳ	27	20	

## 3 讨论

IPASS、OPTIMAL、NEJ002、WJTOG3405、EURTAC等多项临床试验已表明，*EGFR*敏感突变患者可从一代EGFR-TKIs治疗中获益^[9-11]^，三代EGFR-TKI(奥希替尼)针对T790M突变的患者具有显著的疗效。研究表明，不同靶向药物发生耐药的机制不同，如一线EGFR-TKI的主要耐药机制是T790M，而三代EGFR-TKI的耐药机制包括C797S等^[12]^。由于治疗药物引起的时间异质性和可能有新发病灶的原因，指南均建议在靶向药物耐药时对患者进行再次活检^[13]^，明确耐药机制，根据耐药机制选择精准的后续治疗。

本研究发现，再次活检中肿瘤组织标本仍然是主要的标本来源，这可能与本研究单位计算机断层扫描(computed tomography, CT)引导下的细针穿刺项目的开展有关，也与临床医生对于肿瘤组织是金标准的认知有关。再次活检中，血液标本送检的例数增加，这与采用ctDNA检测T790M的可及性和临床研究发现相关，BENEFIT、AURA等多项临床试验已证明血液*EGFR*突变阳性的患者可以从EGFR-TKI治疗中获益^[14, 15]^。

再次活检与治疗前标本相比，基因突变的阳性率有变化，治疗前*EGFR*突变率略高于再次活检标本的*EGFR*突变率(约为4%)，分析原因可能在于携带*EGFR*突变的细胞对EGFR-TKI治疗敏感，治疗导致基因突变阳性细胞数量减少。此外也可能是由于再次活检肿瘤标本取材困难，采用ctDNA进行*EGFR*基因检测的敏感性较差，存在假阴性的情况^[16, 17]^。本研究的数据与以往文献报道相似，王洁等^[18]^采用ARMS法分析了264例患者NSCLC患者的血浆和组织标本，其中54例(20.5%)患者在化疗前为阳性突变，化疗后转变为了野生型；24例(9.1%)野生型的患者，在接受化疗后转变为了突变型。这与我们的研究结果相似，在本研究中5例患者在接受化学治疗前后*EGFR*突变状态发生改变。差异类型分别为S768I转换为G719X+S768I(*n*=1)、野生型转换为突变型(*n*=3，2例19Del、1例G719X)和19Del转换为野生型(*n*=1)。综上所述说明化疗对*EGFR*突变状态是有影响的，因此在化学治疗后，动态监测*EGFR*基因状态是十分重要的。

再次活检中，主要的突变类型为T790M，比例为56.2%，这一结果与以往研究相一致，Zheng等^[19]^采用数字PCR对使用EGFR-TKI药物的NSCLC患者的ctDNA进行检测，发现在117例EGFR-TKI继发耐药的患者中，有47%(55/117)的患者发生了*EGFR* T790M突变，并且在*EGFR* T790M阳性和阴性的继发耐药患者中，年龄、性别、组织学、吸烟史及EGFR-TKI作为几线药物治疗等方面均无明显差异。在另一项Ⅱ期临床试验中^[20]^，共招募60例患者，分别于2个月及肿瘤进展时抽取患者外周血，使用数字PCR检测*EGFR* 19del、L858R及T790M的突变状态，最终有44例患者的肿瘤发生进展，其中行再次活检的35例患者中，发现有66%(23/35)的标本发生了*EGFR* T790M突变；有39例患者通过数字PCR检测ctDNA，有23%(9/39)的患者也发现了*EGFR* T790M突变。在本研究中，一线接受靶向治疗的患者RR和CBR均高于一线接受化疗的患者(RR: 72.9% *vs* 30.7%; CBR: 93.7% *vs* 76.9%)。这个结果与以往研究^[21]^相一致。综上所述，靶向治疗对*EGFR*突变状态是有影响的，*EGFR*突变的患者接受不同治疗方案的临床获益也有差异，因此在患者治疗进展后，监测*EGFR*基因状态的改变，根据*EGFR*基因状态的改变对患者的治疗方案进行调整是很重要的。

本实验具有一定的局限性，如标本数量较少，对于突变影响因素分析时纳入的指标少，未来还需要更多的样本和更细致的分析，发现真实世界再次活检和初次活检标本中的最重要影响因素。

综上所述，一线采用化疗或EGFR-TKI治疗的患者，在真实世界的临床实践中，治疗前和疾病进展时*EGFR*基因突变状态差异较大，因此为了更精准地克服耐药，了解耐药后的肿瘤驱动基因状态，有必要在耐药时进行再次活检，明确*EGFR*基因突变状态。
